# Gallic Acid Based Black Tea Extract as a Stabilizing Agent in ZnO Particles Green Synthesis

**DOI:** 10.3390/nano11071816

**Published:** 2021-07-13

**Authors:** Marta Fiedot-Toboła, Anna Dmochowska, Bartłomiej Potaniec, Joanna Czajkowska, Roman Jędrzejewski, Magdalena Wilk-Kozubek, Ewa Carolak, Joanna Cybińska

**Affiliations:** 1 Łukasiewicz Research Network−PORT Polish Center for Technology Development, Stabłowicka 147, 54-066 Wrocław, Poland; anna.dmochowska@ensam.eu (A.D.); bartlomiej.potaniec@port.lukasiewicz.gov.pl (B.P.); joanna.czajkowska@port.lukasiewicz.gov.pl (J.C.); roman.jedrzejewski@port.lukasiewicz.gov.pl (R.J.); magdalena.wilk-kozubek@port.lukasiewicz.gov.pl (M.W.-K.); ewa.carolak@port.lukasiewicz.gov.pl (E.C.); joanna.cybinska@port.lukasiewicz.gov.pl (J.C.); 2 Faculty of Chemistry, University of Wroclaw, 14 F. Joliot-Curie Str., 50-383 Wroclaw, Poland

**Keywords:** zinc oxide, nanoparticles, black tea extract, gallic acid, green synthesis, antioxidants, antimicrobial activity

## Abstract

In this work, zinc oxide particles (ZnO NPs) green synthesis with the application of black tea extract (BT) is presented. A thorough investigation of the properties of the extract and the obtained materials was conducted by using Fourier transform infrared spectroscopy (FTIR), liquid chromatography-mass spectrometry (LC-MS), X-ray diffraction (XRD), scanning electron microscopy (SEM), thermogravimetric analysis (TGA), and quadrupole mass spectroscopy (QMS). The obtained results indicated that the amount of used BT strongly influenced the morphology, chemical, and crystalline structure of the obtained particles. The investigation demonstrated that the substance present in black tea (BT) extract, which was adsorbed on the ZnO surface, was in fact gallic acid. It was found that gallic acid controls the crystallization process of ZnO by temporarily blocking the zinc cations. Additionally, these organic molecules interact with the hydroxide group of the precipitant. This blocks the dehydration process stabilizing the zinc hydroxide forms and hinders its transformation into zinc oxide. Performed measurements indicated that obtained ZnO particles have great antioxidant and antimicrobial properties, which are significantly correlated with ZnO–gallic acid interactions.

## 1. Introduction

Biosynthesis, or green synthesis of nanoparticles, has been under the radar of many researchers. The various methods following the principles of green chemistry have received a lot of attention due to the low-toxicity of used substrates, their affordability, and inexpensiveness [1]. Metal or metal oxide nanostructures have been synthesized with an application of various extracts prepared from the part of a plant. The extracts are used as either stabilizing or reducing agents. Some of the methods involve using the leaves of plants such as jackfruit (*Artocarpus heterophyllus*) [2] or *Carissa carandas* [3], flowers like *Anchusa italica* [4], the fruits of plants, including *Acacia nilotica* [5], the peels of the fruits (e.g., a banana (*Musa paradisiaca*) peel [6]), or the root of the plant like *Berberis vulgaris* [7].

Among many nanomaterials, zinc oxide nanostructures are exceptionally attractive thanks to their unique functional properties and the diversity of their applications. They have been used in many fields as gas sensors [8], piezoelectric devices [9], solar cells [10], fillers in polymeric nanocomposites [11,12], coatings [13,14], antimicrobial agents [15], and in textiles [16].

Tea infusion is one of the most popular beverages worldwide. The brew is prepared from the leaves of the *Camellia sinensis* plant, which originally comes from China [17]. Depending on the processing of the leaves, the types of tea may be divided into three major groups—green, oolong, and black tea, all of them varying in the fermentation level of the leaves [18]. Green tea is the unfermented one, black tea is completely fermented, while oolong tea stands in the middle, being only partially fermented [19].

The leading components found in tea are polyphenols, which account for 20–35% of dry weight [20]. The fermentation process causes changes in the amount of polyphenols in the leaves due to their oxidative polymerization, leading to the formation of thearubigins and theaflavins, which are responsible for the dark red color of the black tea [21,22]. The polyphenol group is divided into two main categories of components. The first one being the catechins like (−)-epigallocatechin gallate (EGCG), which can constitute for 50–80% of all the catechins in tea. The second group includes the phenolic acids like gallic acid, whose amount is notably increased in black tea due to the processes occurring during fermentation [23,24].

It was reported that except for polyphenols, tea infusions contain many compounds such as polysaccharides, whose amount depends on the maturity of the tea leaf [25]; alkaloids, with caffeine being the easiest one to find in all kinds of tea [26]; amino acids, with theanine taking up to 50% of all free amino acids found in teas [27]; and saponins that have displayed antifungal activity against *Rhizopus stolonifer* [28]. The amount of the various phytochemicals in the leaves is strongly dependent on the processing during the production, the age of the plant, and its origin. In addition, it is difficult to assess the number and nature of the compounds in the tea infusion as it fluctuates with the preparation method (e.g., brewing time) [29].

The leaves of black tea have been used before in order to obtain various metal or metal oxides nanoparticles. The aqueous extract was employed for synthesizing nanoparticles of palladium [30], iron oxide [31], copper oxide [32], and iron, copper, and silver [33]. In most cases, the black tea extract serves as a reducing agent for the precursor of the nanoparticles. In addition, an ethanol black tea extract was applied for the synthesis of gold nanoparticles [34]. Zinc oxide nanoparticles were also synthesized with black tea extract. Therein, the black tea extract served as the reducing agent for hydrothermal syntheses (e.g., to reduce zinc nitrate [35,36,37] or zinc sulfate [38]).

In the presented work, zinc oxide structures were successfully obtained by a green synthesis method in which the black tea water extract was used as a stabilizing agent. This natural compound has not been previously used in this role. Due to the complicated composition of the extract and the ability to stabilize the nanoparticles by its individual ingredients, understanding the mechanism of ZnO formation under these conditions is a very difficult scientific issue. Its explanation was the main subject of this article. Moreover, a very important aspect of this work was the analysis of the relationship between the material properties of the obtained nanoparticles and their possible functionality. Therefore, at first, the chemical composition of the black tea extract was characterized. Then, the chemical and crystalline structure, and morphology of the final materials were thoroughly described and compared with the antioxidant and antimicrobial properties of the synthesized ZnO.

## 2. Materials and Methods

### 2.1. Black Tea Extract (BT) Preparation

A total of 3.00 g of black tea leaves (purchased at the local grocery store) were weighed in a beaker and mixed with 100 mL of distilled water. The mixture was boiled for approximately 5 min at 100 °C (until the color of the extract turned to a very dark burgundy). The mixture was then cooled down and filtered three times. The black tea extract was stored in a refrigerator at 4 °C until further use.

### 2.2. Zinc Oxide Particles Synthesis

Various volumes of the black tea extract were added to the 1 M zinc acetate water solution. The goal was to obtain mixtures with the different ratios (v:v) of the black tea extract versus Zn^2+^ ions as follows: 1:12 (BT1), 1:6 (BT2), and 1:1 (BT3). The solutions were mixed with a magnetic stirrer at room temperature for 10 min. Next, the 1.0 M water solution of sodium hydroxide was added dropwise until the pH increased to 12. The obtained suspensions were left to mix for another 2 h at room temperature. Afterward, the precipitants were washed twice with distilled water and centrifuged (20 min, 6000 rpm/4427 rcf). The washing and centrifuging process was repeated twice. The products were dried at 60 °C for about 24 h until constant mass and ground using a mortar. All reagents were purchased from Sigma-Aldrich (Sigma-Aldrich Co., St. Louis, MO, USA).

### 2.3. Characterization of Black Tea Extract

The chemical composition of the obtained black tea extract was examined by two methods: Fourier transform infrared spectroscopy and liquid chromatography-mass spectrometry.

The FTIR analysis of the functional groups was performed on the dried extract—after water evaporation in 60 °C until constant mass. The sample was ground, mixed with dried spectroscopic grade KBr, and formed into a pellet. The spectrum was recorded in the range of 350–4000 cm^−1^ at 4 cm^−1^ resolution (Tensor 27 EQ spectrometer, Bruker, Bremen, Germany).

LC-MS analysis was executed as follows: right before the measurement, 200 µL of the extract was diluted to 1 mL with ultrapure water and used for LC injection. Hypergrade (LC-MS grade) solvents were used. The calibration mixture was 10 mM NaOH in 1:1 (v:v) water:isopropanol with the addition of 0.2% formic acid. The measurement was performed on high-resolution Q-ToF spectrometer maXis impact (Bruker Daltonics, Bremen, Germany) equipped with an electrospray ionization (ESI) source and connected to a Dionex UltiMate 3000 RSLC (Thermo Scientific, Waltham, MA, USA) ultrahigh-performance liquid chromatograph. The chromatographic separations were carried out on Syncronis C18 100.00 × 2.10 mm × 1.70 μm column (Thermo Scientific, Waltham, MA, USA). All reagents were purchased from Sigma-Aldrich (Sigma-Aldrich Co., St. Louis, MO, USA). The data were analyzed with Data Analysis 4.1 software (Thermo Scientific, Waltham, MA, USA). The extracted ion chromatograms (EICs) for every ion that gave good quality fragment spectrum were generated semi-automatically with a manual check of every chromatographic peak generated that way. For each EIC peak, the molecular formula of the parent ion was generated using the SmartFormula algorithm with maximum admissible error of 5 ppm (10 ppm in case of no valid formulas in 5 ppm range). Then, MS/MS spectra for every EIC peak were compared against METLIN and NIST 11 spectral databases. For every compound identified that way, a specific record was created. In the case of most of the chromatographic peaks, we were not able to get a clear hit on the database, so we used literature data on tea extract analysis for manual identification of certain compounds based on retention order, fragment spectra comparison, etc. [39,40]. For the remaining peaks that were not identified by the database and literature search, we performed manual fragment spectra annotation and came up with the most probable identification or partial identification. 

### 2.4. Characterization of ZnO Particles

The X-ray diffraction analysis (Empyrean, Malvern PANalytical, Malvern, UK) was performed to determinate the phase composition, crystallite size, and strain. The following parameters were set: Cu-Kα radiation (λ = 1.54 Å), operating voltage—40 kV, current 30 mA, 2Θ range 10–100°, step 0.007°, scan speed 150 s/step (Pixcel detector). The X’Pert HighScore Plus program with ICDD PDF-4+ 2019 database was used to identify the phase composition of the samples. The zinc oxide crystallite size and lattice strain were calculated by using a line profile analysis (LPA in HighScore Plus, Malvern PANalytical, Malvern, UK) according to the Williamson–Hall method (1) [41].

(1) $$\beta_{\mathrm{hkl}}\cos\left(\theta \right)=\frac{k\lambda }{D}+4\varepsilon \sin$$

where β is the peak width at half maximum (FWHM) [rad]; k is the Scherrer constant (0.9); λ is the wavelength of Cu-Kα radiation; D is the crystallite size; and ε is the lattice strain.

The morphology of the ZnO powders was determined by scanning electron microscopy analysis (SEM) using a dual beam microscope (Helios 450HP, Nanolab Technologies Inc, Milpitas, CA, USA ). The samples were placed directly on a carbon tape without coatings and imaged at a low-voltage mode (≤5 keV). The approximation of the obtained particle dimensions was based on dimensioning from a series of SEM images and their averaging.

The FTIR spectroscopy was used to analyze the functional groups in the materials. The measurement parameters were the same as for the analysis of the black tea extract.

Thermogravimetric analysis was performed to determine the powders’ thermal stability (TGA2 thermogravimetric analyzer, Mettler Toledo, Columbus, OH, USA). The measurements were conducted using alumina crucibles (~5.00 mg) in an air atmosphere (30 mL/min) in the range of 25–600 °C with a 10 °C/min heating rate. Additionally, the Vyazovkin free kinetics model (2) [42] was used to determine the activation energy (E_a_) of the degradation process. For this purpose, the TG measurements were repeated in the same conditions but with different heating rates (5, 15, 20 °C/min).

(2) $$\frac{d\alpha }{\mathrm{dt}}=\mathrm{Aexp}\left(\frac{-E}{\mathrm{RT}}\right)f\left(\alpha \right)$$

where R is the gas constant; a is the slope of linear plot; t is the time; a is the constant; T is the temperature.

The analysis of the gases evolved during the decomposition of the ZnO particles was done by using the STA 449 F1 Jupiter Netzsch thermal analyzer coupled with a quadrupole mass spectrometer QMS Aëlos 403D (Netzsch, Selb, Germany) and Tensor 27 EQ spectrophotometer (Bruker, Bremen, Germany). The sample that was synthesized with the largest amount of black tea extract was heated up to 600 °C with the rate of 10 °C/min in the nitrogen atmosphere (50 mL/min).

The antioxidant capacity of the black tea extract and ZnO particles was determined using the 2,2′-azino-bis(3-ethylbenzothiazoline-6-sulfonic acid (ABTS) assay. This spectrophotometric assay is based on the ability of the antioxidant substance to quench the colored free ABTS radical cation [43]. The blue-green colored ABTS radical cation, formed by the direct reaction of ABTS with potassium persulfate, has absorption maxima at 415, 645, 734, and 815 nm. The quenching of the free ABTS radical cation results in a decrease in absorbance at the selected wavelength, which was visualized by discoloration of the ABTS radical cation. The degree of discoloration of the ABTS radical cation measured over time depends on the concentration of the antioxidant substance and the duration of the reaction, so that for a fixed reaction time, the antioxidant activity can be expressed as a dependence of the degree of discoloration on the concentration of the reference substance 6-hydroxy-2,5,7,8-tetramethylchroman-2-carboxylic acid (Trolox). Trolox is a water-soluble analogue of vitamin E with high antioxidant activity, commonly used as the reference substance. All reagents were purchased from Sigma-Aldrich (Sigma-Aldrich Co., St. Louis, MO, USA).

ABTS working solution was obtained by mixing equal volumes (1.0 mL) of the 14.0 mM ABTS stock solution with the 5.0 mM potassium persulfate stock solution in a 200 mL flask. The mixture was stored in the dark at room temperature for 16 h and then diluted with distilled water to a volume of 200 mL. Trolox standard solutions with final concentrations in the range of 0–10.0 μM were prepared by a series of dilutions of the 8.1 mM Trolox stock solution. Sample solutions were obtained by placing 0.10 g of the sample in a 10.0 mL flask and its dilution with distilled water to a volume of 10.0 mL. Solutions containing ZnO particles were centrifuged at 6000 rpm/4427 rcf for 2 min. A total of 50.0 μL of Trolox standard or the sample solution were added to 4.0 mL of the ABTS working solution in a 1 cm light-path polystyrene cuvette. Absorbance values were read at 734 nm before adding and 10 min after adding and mixing the content of the cuvette. For this purpose, a Thermo Scientific Evolution 300 UV–Vis spectrophotometer (Thermo Fisher Scientific, Waltham, MA, USA) was used. Appropriate solvent blanks were run for the Trolox standards and the sample solutions. All determinations were carried out at least two times. The antioxidant activity results are given as TEAC values (mM Trolox).

The antimicrobial activity of the synthesized samples was tested against Gram-positive bacteria (*Staphylococcus aureus*—ATCC 6538), Gram-negative bacteria (*Pseudomonas aeruginosa*—ATCC 9027), and yeast (*Candida albicans*—ATCC 10231). An overnight broth culture of each strain was used to prepare inoculum. The suspensions of bacteria and yeast were adjusted to a density of 0.5 and 0.6 McFarland standard, which is 1.5 × 10^8^ CFU/mL. Then, the cultures were diluted 100-fold into 10.0 mL of Mueller–Hinton Broth (Biomaxima) medium. The synthesized powders (BT1, BT2, BT3) were added to the respective cultures to a final concentration of 10 mg/mL. Cultures were incubated at 37 °C with shaking at 170 rpm and samples were collected at 2, 6, and 24 h. Samples were immediately diluted in normal saline (0.9%). The number of colony-forming units was determined using the Miles and Misra technique [44,45,46]. Briefly, TSA (trypticase soy agar) plates were divided into eight equal sectors, labeled with the dilution from 100 to 10^−7^. In each sector, 3.0 × 10.0 µL of the appropriate dilution was dropped onto the agar surface. The plates were incubated at 37 °C for 24 h. The images of plates after a certain time of incubation were presented on the example of the BT1 sample and *S. aureus* bacteria (Figure S1). The CFU/mL and the percentage of the viability reduction was counted. The assay was done three times independently.

## 3. Results and Discussion

### 3.1. Black Tea Extract Spectral Analysis

As mentioned before, the chemical composition of a tea extract is very complex. It was reported that it consists of about four thousand bioactive compounds like polyphenols, phenols, phenolic acids, alkaloids, amino acids, carbohydrates, proteins, chlorophyll, and volatile organic compounds. The composition is not constant and depends on various factors, with the most important being the origin and age of the plant, and the method of preparation [47,48,49]. For these reasons, the exact composition always has to be confirmed.

The FTIR results indicated many peaks on the spectrum of black tea. These were associated with different functional groups: –OH (~3400 cm^−1^), –CH (~2920 cm^−1^), COOH (1697 cm^−1^), COO^−^ (1697, 1403 cm^−1^), –CH_2_ and CH_3_ (1448, 1372 cm^−1^), C–O–C (1231, 1030 cm^−1^), –C–O (1145 cm^−1^), aromatic ring (823, 758, 708 cm^−1^), and –OH in phenols (612 cm^−1^) (Figure S2). These attributions were made thanks to the literature data [47,48,49], however, due to the occurrence of similar functional groups in the mentioned compounds, it is very difficult to distinguish and name them only on the basis of these results.

To perform a detailed investigation of the chemical composition of the obtained black tea extract, LC-MS analyses were conducted. In total, 48 compounds were detected in both ion modes (Table S1). It was shown that the largest group of ingredients in black tea infusion were flavonoids, both aglycones and glycosides, from the flavan-3-ols (e.g., quercetin, kaempferol) and catechin (e.g., epicatechin, catechin, theaflavin) groups, constituting over two thirds of the identified compounds. Ten of the identified compounds were acids, mainly quinic acid, gallic acid, and *p*-coumaroylquinic acids. Moreover, the analysis also showed the presence of amino acids and an alkaloid (e.g., theanine and caffeine, respectively) [39,40].

### 3.2. Characterization of ZnO Particles

#### 3.2.1. Chemical Structure

**Figure 1:**
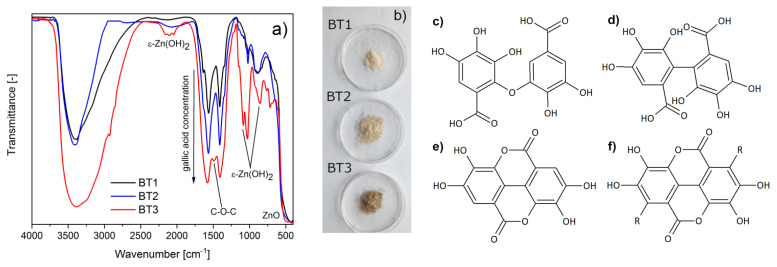
(**a**) FTIR spectra of the samples; (**b**) images of the samples; gallic acid autoxidation products: (**c**) C–O dimer, (**d**) C–C dimer, (**e**) ellagic acid, (**f**) gelate-based polymer.

The FTIR measurements of the obtained samples confirmed a successful synthesis of ZnO particles, which was evident by the presence of Zn–O oscillation (~480 cm^−1^). Additionally in the BT3 sample, the peaks characteristic for zinc hydroxide were observed (triplet ~2100, doublet ~1050, and 850 cm^−1^), which were absent in the spectra of another measured materials. In addition, some oscillations connected with organic compounds of black tea extract were observed. They could be assigned to appropriate functional groups: –OH (~3400 cm^−1^), C–H (~2900 cm^−1^), COO^−^ (~1576, 1406 cm^−1^), C–OH (~1028 cm^−1^), and aromatic ring (~880 cm^−1^). The more black tea extract was used during ZnO synthesis, the more intensive these peaks were. This suggests that a larger amount of organic components was adsorbed on the zinc oxide surface (Figure 1a). This was also observed by a gradual color change within the samples from cream to brown (Figure 1b). Taking into account the spectroscopy analysis (FTIR, LC-MS) of the black tea extract and literature data [50,51,52], it could be concluded that the component adsorbed on the surface of ZnO was gallic acid. COO^−^ groups were observed, so it could be supposed that zinc ions present on the ZnO surface can chemically interact with gallic acid. Taking into account the FTIR results and the literature data, this occurred most probably by complexing the metal ions with the hydroxyl groups of the acid [53,54] or carboxylic group. Additionally, in BT3, a new peak was observed at about 1480 cm^−1^, which could be connected to C–O–C oscillations in esters (Figure 1a). It is supposed that gallic acid particles could interact not only with metal ions, but also between each other because of the autoxidation reaction. Other authors have suggested that as a result of this process, C–O (Figure 1c) or C–C (Figure 1d) bonded, ellagic acid (Figure 1e), or gallate-based polymers (Figure 1f) can occur [55,56,57]. The obtained FTIR results showed C–O–C bonding in the BT3 sample, which suggests that the C–O dimer, ellagic acid, or gellate-based polymer could be obtained.

#### 3.2.2. Crystalline Structure

**Figure 2:**
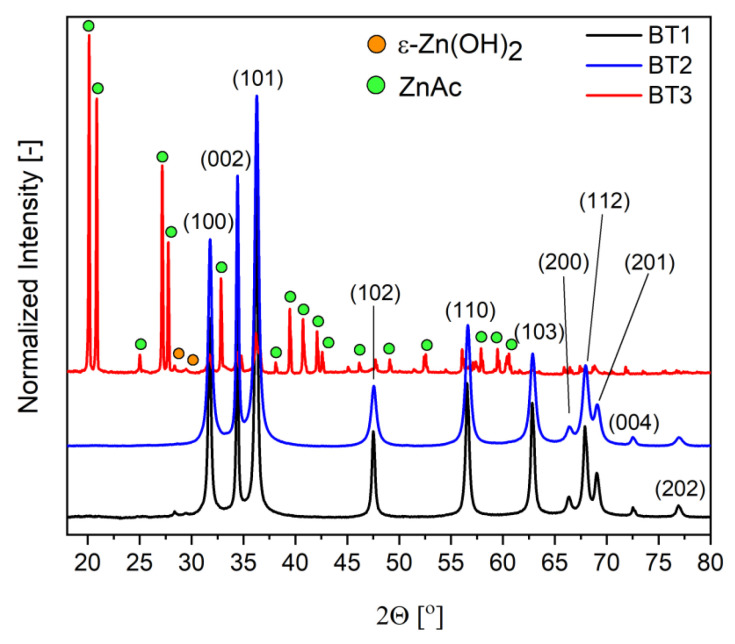
Diffractograms of the samples.

The diffractograms of the samples were different for each material. In the case of BT1 and BT2, only the wurtzite-type zinc oxide (ICDD 01-070-8070) was observed. In BT3, a hexagonal form of ZnO was also present, but the dominant one was wülfingite, which is a crystalline form of zinc hydroxide with orthorhombic unit cell (ε-Zn(OH)_2_) (ICDD 04-012-2300). Additionally, a very small amount of zinc acetate (ZnAc) (ICDD 00-021-1467) fraction was indicated (Figure 2).

The authors obtained similar results when pectin was used as a stabilizing agent in the course of the synthesis of ZnO particles [6]. To understand the possible influence of black tea extract on zinc oxide crystallization, its mechanism has to be described. In the first step, the zinc ions were formed because of zinc acetate dissociation. After adding sodium hydroxide into the reaction mixture, the OH^−^ anions were created, which could interact with zinc cations. As a result, amorphous zinc hydroxide will be obtained, which could further transform into crystalline ε-Zn(OH)_2_ and then into ZnO.

From the literature data, it is known that gallic acid has a great tendency to metal ion chelation [53,58,59]. For this reason, it could slow down the hydroxide precipitation process by temporarily blocking the cations. With the addition of hydroxide, the pH of the system gradually changes and the equilibrium state of the system shifts toward the formation of an amorphous and crystalline hydroxide phase. Thanks to that phenomenon, the crystallization process is controlled at this stage. Additionally, it is known that recrystallization of ε-Zn(OH)_2_ to ZnO occurs not by dissolution and liquid phase reactions, but by solid phase processes. The dehydration of the hydroxide begins inside the crystal and gradually progresses toward the outside. Therefore, the hydroxide residues are very often found on the surface of the final zinc oxide [60]. The obtained XRD results suggest that gallic acid can stabilize the hydroxide form and hinders its transformation into zinc oxide. This is evident by the fact that in samples (BT1, BT2) with the smaller amount of this organic component, only the wurzite-type ZnO was observed on their surface. In the case of BT3, a very small amount of ZnO in the BT3 sample and very well crystallized ε-Zn(OH)_2_ were present. These conclusions were also supported by FTIR results. Additionally, crystallite size (D) and microstrains (ε) of zinc oxide crystals were calculated using the Williamson–Hall equation [41]. The obtained results showed that in the case of the BT1 and BT3 samples, the D values decreased (23, 19 nm) and the ε values increased (0.04, 0.23) as a function of black tea concentration. In the BT3 sample, ZnO diffraction peaks were not intense enough to count these values as ε-Zn(OH)_2_ was the dominant crystalline fraction. These observations could suggest that organic molecules adsorb on the particles’ surface, which causes the disturbance in the decreasing size of the crystal structure of ZnO. Taking into account the literature data, most probably gallic acid interacts with the hydroxide group, which is blocking the dehydration process and stabilizes the ε-Zn(OH)_2_ form [56]. The lack of a linear relationship between the amount of organic stabilizing agent and the amount of ε-Zn(OH)_2_ suggests that there is a minimal concentration of gallic acid that is needed to block the transformation of crystalline hydroxide to oxide.

#### 3.2.3. Morphology

**Figure 3:**
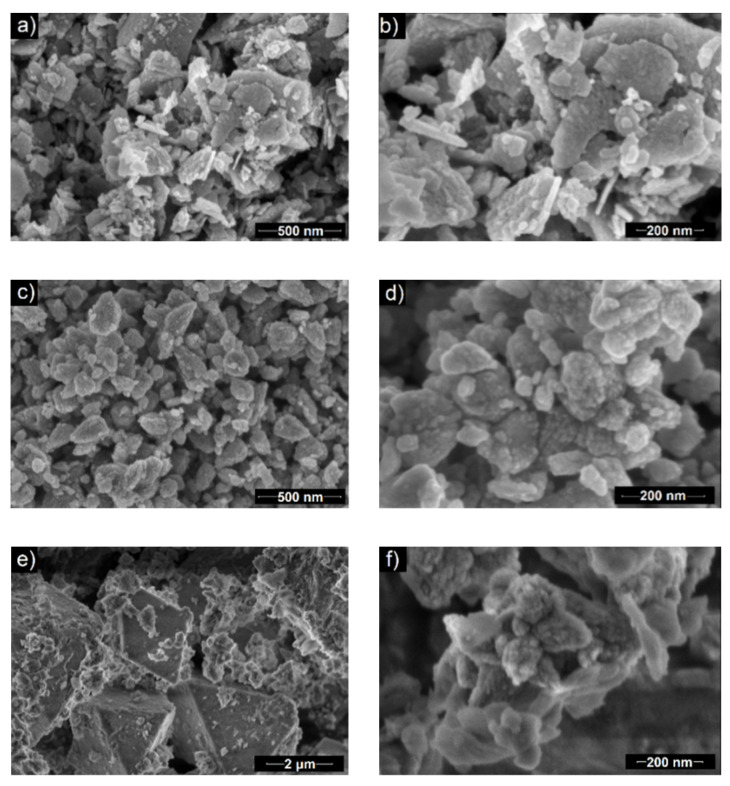
SEM images of the samples: (**a**,**b**) BT1, (**c**,**d**) BT2, (**e**,**f**) BT3.

Scanning electron microscopy indicated that the morphology of obtained ZnO particles depends on the amount of used black tea extract. In the case of the BT1 sample, the particles were in the form of nanoflakes, whose lengths and widths were around 100–550 and 21 nm, respectively (Figure 3a,b). BT2 particles had a nanocone-like shape (50–300 nm length and 30–175 nm width), which were agglomerates of smaller units (diameter ~14 nm) (Figure 3c,d). In the image of the BT3 sample, two kinds of structures were visible. One were small, quasi-spherical with a diameter of ~150 nm. The other structure had octahedral morphology and were much bigger with the side length around 3.5 µm (Figure 3e,f). It was supposed that they were separate fractions that may have a different material composition. The amount of octahedral structures were greater than the spherical ones. Based on the XRD and FTIR results, it could be concluded that small particles are made of hexagonal type ZnO crystals and octahedral are built from orthorhombic ε-Zn(OH)_2_ [60,61,62]_._

Generally, it could be assumed that by increasing the amount of black tea extract, the particles change their shape and size starting from flat and big particles to more spherical and smaller. Additionally, in the BT3 sample, a fraction of a new material was obtained. Based on previous work by the authors [6], it was supposed that chemical components of the tea extract interact with ZnO crystallites. First, they adsorbed on the wurtzite polar surfaces (0001 and 000$\bar{1}$) and suppressed the growth along the c-axis [63]. By increasing the amount of organic compounds, they could start to adsorb on each side of the ZnO hexagonal unit cell, which inhibits its growth in every direction. Because the wülfingite fraction was observed in the BT3 sample, it could be concluded that the black tea extract may influence the process on each of its steps. This was reflected in an altered crystal growth and formation of various phases in the final product.

#### 3.2.4. Thermal Analysis

**Figure 4:**
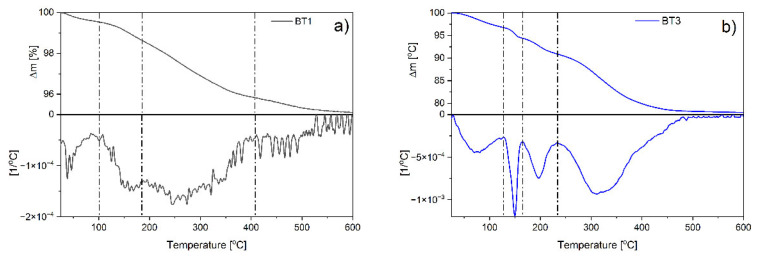
TG and DTG curves of: (**a**) BT1; (**b**) BT3 samples.

Thermal stability of the samples obtained with the smallest and the biggest amount of the black tea extract was examined by thermogravimetric analysis. The results indicated that in the case of BT1, four mass losses were observed with the maximum degradation rates found at around 40, 160, 270, and 450 °C (Figure 4a). The observations for the BT3 sample were definitely different. Four degradation stages were also observed, however, the maxima were found at other temperatures: 80, 150, 195, and 330 °C (Figure 4b).

Based on FTIR, XRD, SEM analysis, and the authors’ previous data [6], it could be supposed that moisture evaporation can be observed during the first step. Then, at around 150–160 °C, degradation of amorphous zinc hydroxide was observed. Further temperature increase caused ε-Zn(OH)_2_ decomposition (~195 °C). Finally, the degradation of organic components occurs in different ways depending on the sample. In the case of BT1, the process was multi-stage and occurred at temperatures typical for gallic acid decomposition [53], which proves that the compound was adsorbed on the ZnO surface. In BT3, one stage was observed that took place at higher temperatures than BT1. This could confirm the conclusion postulated in FTIR studies about the interactions of gallic acid–gallic acid, which increase the thermal stability of the organic phase present on the ZnO surface.

Comparison of the values of individual weight losses were found to be significantly greater for the BT3 sample than for the BT1 at each degradation stage. The weight loss associated with moisture has increased from 0.46 to 3.23% and the loss connected with amorphous zinc hydroxide from 0.91 to 2.38%. ε-Zn(OH)_2_ was present only in BT3 (3.48%). The gallic acid content was about 3.52% in BT1 and 13.45% in BT3 (Figure 4).

In summary, the TG results suggest that the more black tea extract was used, the more gallic acid molecules were adsorbed on the surface of the particles. This led to an increase in their tendency to adsorb moisture and block the transformation of both amorphous and crystalline zinc hydroxide to zinc oxide. Additionally, a large amount of gallic acid particles provokes their chemical interactions with each other, which was supported by the FTIR results.

**Figure 5:**
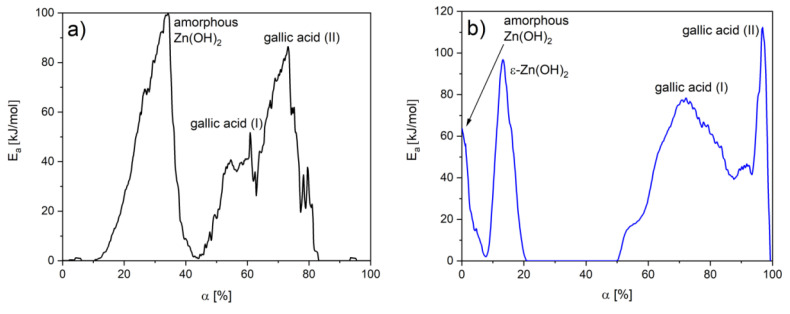
E_a_ changes as a function of conversion degree in the sample: (**a**) BT1, (**b**) BT3.

In order to specify the interactions between gallic acid particles, the values of activation energy were determined as a function of materials conversion. In the case of BT1, the E_a_ value of amorphous Zn(OH)_2_ was higher than in BT3. Supposedly, this fraction exhibits a stronger bond with the ZnO surface. In the BT3 sample, the degradation energy of ε-Zn(OH)_2_ was also detected. In both cases, the decomposition of gallic acid is a two-step process, but the E_a_ values were higher in the BT3 sample. This was not noticeable on the thermogravimetric curves due to an overlap of processes at similar temperatures in the BT3 sample. These observations could indicate gallic acid–gallic acid interactions (Figure 5).

**Figure 6:**
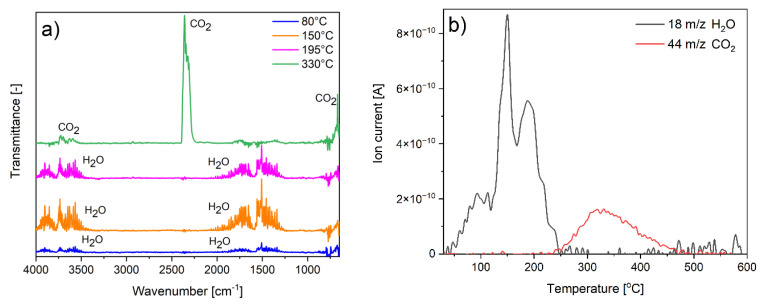
Spectra of evolving gaseous products: (**a**) FTIR; (**b**) QMS.

The spectral analyses of gas products evolving during decomposition were conducted to confirm the composition and the proposed degradation mechanism. Because of the largest amount of the additional fraction, the BT3 sample was tested. Both FTIR and QMS spectra showed that during the first three degradation steps, only water was detected. In the last step, only carbon dioxide was produced (Figure 6). This confirms the previous results obtained from other measurements and points to the proposed sample composition and its degradation mechanism.

#### 3.2.5. Antioxidative Properties

Black tea extract owes its antioxidant properties mainly to the presence of polyphenols [64]. Its concentration depends on many factors related to the processing of the leaves but also to the process of their infusion including the pH of the water used, the temperature, and the infusion time [65]. In this study, the antioxidant activity of black tea extract was calculated as 453.70 mM Trolox based on the ABTS assay. The obtained result was within the range of values found in the literature [66]. The obtained particles also showed antioxidant properties with values of 17.77, 43.60, and 63.32 mM Trolox for BT1, BT2, and BT3 samples, respectively. These results could be connected with the increasing concentration of the gallic acid on the ZnO surface in subsequent attempts, as demonstrated by the FTIR and TG measurements, which is most likely a result of the adsorption of gallic acid on the surface of particles. Based on the thermal analysis data, the gallic acid content was about 3.52% in BT1 and 13.45% in BT3. The difference of these concentrations between the BT3 and BT1 samples was about four times, which showed the same tendency as an increase in antioxidant activity and confirms the presented hypothesis.

As mentioned, many polyphenols exhibit antioxidant properties. This is connected with five main mechanisms: hydrogen atom transfer (HAT), single electron transfer (SET), sequential proton loss electron transfer (SPLET), sequential double proton loss electron transfer (SdPLET), or radical adduct formation (RAF). In all of them, the polyphenols play the role of a donor. The preferred process depends on the dissociation enthalpy of the hydroxyl group and the ionization potential of a measured polyphenol molecule [54]. The reaction environment also plays a crucial role. In aqua solutions, SPLET or SdPLET are dominant for free radical scavenging while in an anhydrous environment, HAT and RAF take place [67]. Gallic acid in its basic form is not the most active natural polyphenol. However, in the case of ionization of the carboxyl group, the enthalpy of hydroxyl groups is significantly reduced, which gives gallic acid the strongest antioxidant activity [54]. FTIR results showed that this form of the organic molecule was present in the measured particles. Additionally, it is known that in free radical scavenging, the most effective position of –OH group is the *para* position. The hydroxyl groups in *meta* positions stabilize formed radicals, which increase antioxidant capacity [68]. These two phenomena are most likely responsible for the high antioxidant activity of the obtained samples.

The differences between the activities of the particles could be connected not only with increasing gallic acid concentration between the samples, but also the opposite effect of ZnO. Similar conclusions were also demonstrated by other authors [69]. This oxide is inherently very non-stoichiometric, so its crystal structure may contain numerous defects such as interstitial oxygen or oxygen vacancies. Thanks to the presence of oxygen vacancies, depending on the environment, atmospheric oxygen or water molecules could be very easily adsorbed on the ZnO surface in the form of ions or radicals [8]. Due to this phenomenon, this material has oxidizing properties [15,70], which could lower the antioxidant capacity of the samples.

#### 3.2.6. Antimicrobial Assay

**Figure 7:**
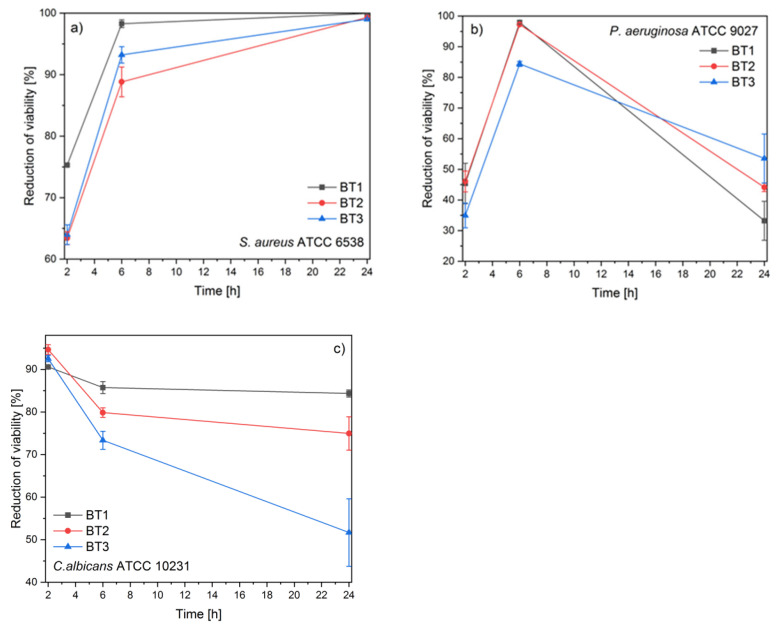
Antimicrobial activity of ZnO NPs against: (**a**) *S. aureus*, (**b**) *P. aeruginosa*; (**c**) *C. albicans*.

The antimicrobial activity of the tested compounds was examined against two bacterial and one fungus strain. The reference strains of *Staphylococcus aureus* ATCC 6538–grape-like Gram-positive cluster, rod shaped Gram-negative *Pseudomonas aeruginosa* ATCC 9027, and fungus *Candida albicans* ATCC 10231 were used for the experiment. The antimicrobial activity was assessed by time-kill assays (Figure 7). The tested compounds showed diversified antimicrobial activity.

The Gram-positive strain was the most susceptible to all samples. The results showed that the reduction of *S. aureus* viability was increasing as a function of time. After 2 h, a very similar activity was observed in the case of BT2 and BT3, but the best effect was displayed by the BT1 sample. After 24 h of exposition, all materials exhibited a bactericidal effect (reduction of viability above 99%) (Figure 7a).

The obtained results for *P. aeruginosa* varied the most during the time of the experiment. At the beginning, the best activity was demonstrated by BT1 and BT2. The strongest antibacterial effect against *P. aeruginosa* was observed after 6 h post-inoculation and significantly decreased afterward. After 24 h, it was visible that the more gallic acid on the ZnO surface, the better the antibacterial activity was (Figure 7b).

The observed high antifungal activity decreased during the time of the experiment. The smallest decrease in the antifungal activity was observed for BT3. It was clearly visible that the reduction of viability decreased as a function of gallic acid concentration (Figure 7c).

Many aspects can cause the antimicrobial activity differences among the ZnO NPs. The most important seems to be the chemical composition, size, and morphology of the used active material and also the microbial structure [1,71].

Zinc oxide is a very popular antibacterial and antifungal agent. Many authors have investigated the mechanism of its activity. It was postulated that the phenomenon is correlated with zinc ion release or free radical generation. The previous studies from the authors indicated that zinc hydroxide is responsible for ion formation and zinc oxide for ROS generation. The comparison of this observation with antimicrobial results clearly demonstrated that not ions, but ROS generation, is more responsible for ZnO biological activity [15]. Considering those, it could be supposed that the decrease in antimicrobial ability as a function of gallic acid concentration is connected with increasing zinc hydroxide content in the samples.

Additionally, the properties of gallic acid have to be considered. Because the black tea extract was used to obtain ZnO particles, its biological activity was determined. It was found that it did not reduce the growth of the tested microorganisms. However, in the literature data, it was demonstrated that pure gallic acid, due to its high tendency to ion and free radical binding, can interact with the microbial cell surface, which leads to a change in its hydrophobicity and charge. In the case of fungus, this organic molecule can interfere with 1,3-β-glucan and ergosterol synthase. All these processes cause a spill out of the cytoplasmic content [72].

To understand the antimicrobial activity of the measured samples, the ZnO–gallic acid interactions have to be taken into account. As mentioned before, the action of zinc oxide is mainly based on the generation of free radicals. Since gallic acid has strong antioxidant properties, the effect may be reduced. On the other hand, these components could show a synergistic effect thanks to the different action mechanisms.

In these studies, three microorganisms with significant differences in the cell wall structure were chosen.

Gram-positive bacteria have a thinner cell wall and are more sensitive to oxidative stress. The tested compounds showed a bactericidal effect against reference *S. aureus*, but the certain mechanisms of action are still unknown, although numerous studies pointing out the antibacterial effect of ZnO based compounds in the production of increased levels of ROS [73]. Since the activity of the tested samples is inversely proportional to the amount of gallic acid, it was suspected that it stabilizes the free radicals present on the ZnO surface by reducing their activity. Therefore, it takes longer to observe ROS interaction with the bacterial cell wall. In the presented studies, *S. aureus* exhibited the highest sensitivity to the obtained samples. These results are comparable with other research groups, which demonstrated that ZnO is more effective against Gram-positive strains [74]. Additionally, the bactericidal effect of the samples presented in this article was observed using a lower concentration of active material than that of the other authors [75] and its value is uncommon for particles of comparable sizes [71].

In contrast, *P. aeruginosa* is a Gram-negative strain that is known for its high resistance to antiseptic and other antimicrobial agents. This is probably due to its low outer membrane permeability. *Pseudomonas* has express specific channel proteins to nutrient incorporation and does not have general diffusion porins [76]. Despite the almost bacteriostatic activity after the first 6 h of the experiment, the persisted cells started to multiply and the antimicrobial activity decreased significantly. The observed decline in reduction of the viability was probably caused by *P. aeruginosa* cell wall structure and its properties. However, it has been shown that *P. aeruginosa* possesses antioxidant defenses including catalase production, which can increase its resistance to ROS produced during the activity of tested compounds [77,78]. After a long time of interaction between this Gram-negative strain and measured samples, their activity increased with gallic acid concentration. This could suggest that despite the start of building resistance against the action of free radicals, the used polyphenol may additionally connect with the cell wall and disrupt its proper functioning. Due to the highest resistance of Gram-negative strains toward antimicrobials, the obtained samples showed only an inhibitory effect on *P. aeruginosa* growth. The presented results were comparable with another author’s study where the inhibitory effect was also observed using a similar concentration of ZnO [79].

Additionally, a fungistatic effect of the synthesized ZnO particles against *C. albicans* was observed. Based on the recent review, there were only a few studies on the antifungal activity of ZnO [71]. A similar analysis conducted by another author showed a comparable inhibitory effect after using a slightly smaller amount of the active ingredient [74]. This proved a high antimicrobial potential on the zinc oxide particles presented in this article. The different concentration used by other researchers needed to obtain a similar effect was likely due to the smaller particle size used in that test. Furthermore, *C. albicans*, as a representative of fungus, has a more complicated cell structure than bacteria. They are eukaryotic cells that have a uniquely composed two-layered cell wall structure. The main components are β-glucans, chitin, and mannoproteins [80], although ergosterol is one of the basal components of the *C. albicans* cell membranes responsible for its integrity [81]. One of the antifungal strategies described in the literature is to develop drugs that are binding to sterols that are present in the fungal cell membranes, leading to cell lysis [82]. A recent report indicated that gallic acid antifungal activity is associated with disturbing the ergosterol synthesis pathway, however, this needs further investigation for *C. albicans* species [54]. In this case, both the free radical production by zinc oxide and the interaction of gallic acid with the cell wall could generate the apoptosis process [83,84]. The obtained long-term results showed that fungi are much more susceptible to the generation of free radicals than the action of organic acid. Therefore, the capture of ROS by gallic acid weakens the biological activity of the tested samples.

## 4. Conclusions

The conducted studies demonstrated that a water black tea extract could be successfully used as the stabilizing agent during ZnO synthesis. The used natural extract consists of 48 organic ingredients. However, it was indicated that it was gallic acid that played a major role in the particle formation at many stages of the process.

First, it controls the crystallization process of ZnO by zinc ion chelation, which disturbs the hydroxide precipitation process by temporarily blocking the cations. Furthermore, the addition of NaOH to the reaction environment shifts the equilibrium state of the system toward the formation of an amorphous and crystalline hydroxide phase. Second, gallic acid molecules can be adsorbed on the particle’s surface by interacting with their hydroxide group. Thanks to that, gallic acid controls the crystallite size, stabilizes the hydroxide form, and hinders its transformation into zinc oxide.

Analysis of the antioxidant properties indicated that they are proportional to the amount of the gallic acid molecules that were on the surface of ZnO particles, but are also correlated with oxidizing properties of ZnO.

In addition, in the case of the antimicrobial activity of obtained samples, the ZnO–gallic acid interactions seem to be crucial. On one hand, their presence may weaken each other’s action, and on the other hand, act synergistically. This depends mainly on the type of the tested microorganisms and the duration of the action of the active agent.

## Data Availability

The data is included in the main text and the supplementary materials.

## Supplementary Materials

The following are available online at https://www.mdpi.com/article/10.3390/nano11071816/s1, Figure S1. Images of TSA plates after culturing and incubation with *S. aureus* ATCC 6538 at 37 °C for 24 h; Figure S2. FTIR spectrum of the black tea extract; Table S1: LC-MS results.
