# Supplementary material for: Gallic Acid Based Black Tea Extract as a Stabilizing Agent in ZnO Particles Green Synthesis
Source: Nanomaterials (Basel). 2021 Jul 13;11(7):1816. doi: 10.3390/nano11071816 (PMC8308264; doi:10.3390/nano11071816)
Supplement: Supplementary file 1 [file nanomaterials-11-01816-s001.zip › nanomaterials-1289105-supplementary.pdf]

Supporting Information for

Gallic Acid Based Black Tea Extract as A Stabilizing Agent in ZnO Particles Green Synthesis

Marta Fiedot-Toboła[1,*], Anna Dmochowska[1], Bartłomiej Potaniec[1], Joanna Czajkowska[1], Roman Jędrzejewski[1], Magdalena Wilk-Kozubek[1], Ewa Carolak[1] and Joanna Cybińska[1,2]

[1] Łukasiewicz Research Network–PORT Polish Center for Technology Development, Stabłowicka 147, 54-066 Wrocław, Poland; anna.dmochowska@ensam.eu (A.D.); bartlomiej.potaniec@port.lukasiewicz.gov.pl (B.P.); joanna.czajkowska@port.lukasiewicz.gov.pl (J.C.); roman.jedrzejewski@port.lukasiewicz.gov.pl (R.J.); magdalena.wilk-kozubek@port.lukasiewicz.gov.pl (M.W-K.); ewa.carolak@port.lukasiewicz.gov.pl (E.C.); joanna.cybinska@port.lukasiewicz.gov.pl (J.C.)

[2] Faculty of Chemistry, University of Wroclaw, 14 F. Joliot-Curie Str. 50-383 Wroclaw, Poland

* Correspondence: marta.fiedot-tobola@port.lukasiewicz.gov.pl; Tel.: +48-71-734-7154

a)
b)
c)
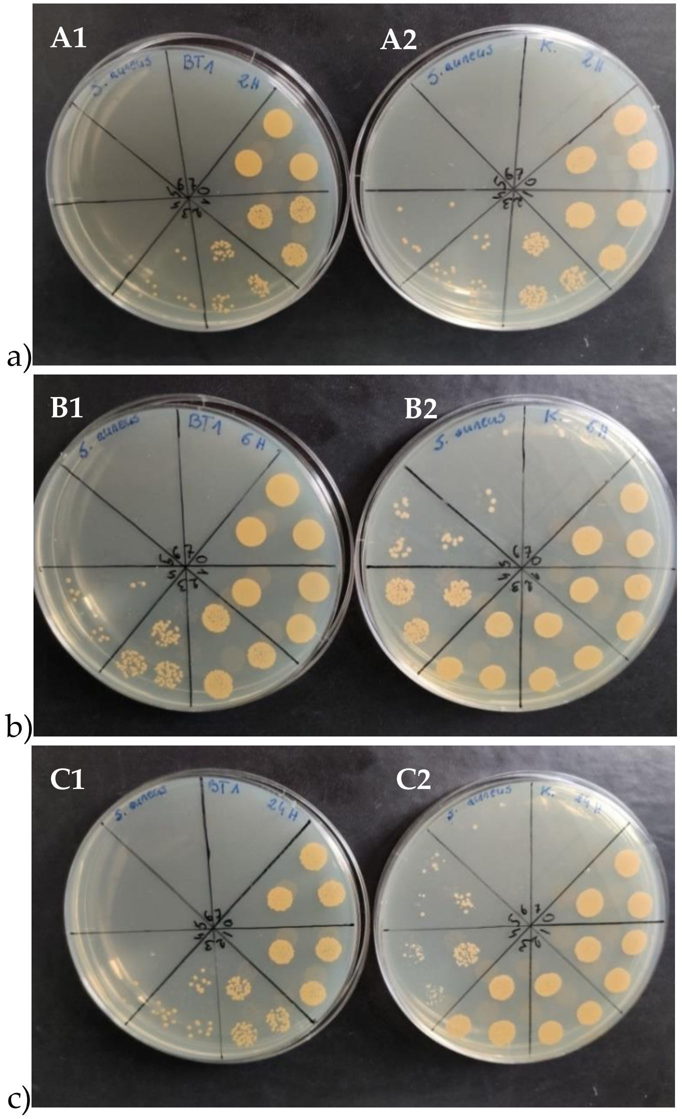

Figure S1. Images of TSA plates after culturing and incubation with *S. aureus* ATCC 6538 at 37°C for 24 hours: a) A1: plate after 2h of treatment BT1, A2: control plate, b) B1: plate after 6h of treatment BT1, B2: control plate, c) C1: plate after 24h of treatment BT1, C2: control plate.

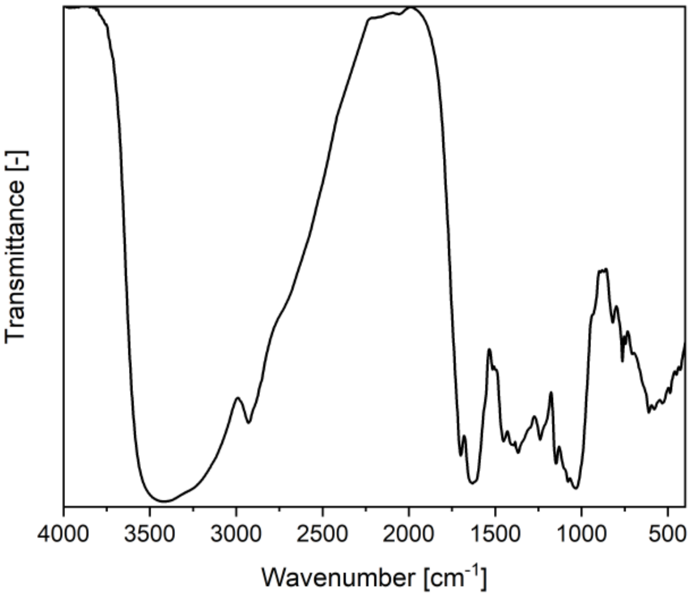

Figure S2. FTIR spectrum of black tea extract.

Table S1. Data obtained from LC-MS experiments.

Negative ion mode								
No.	m/z [measured]	Identification	Molecular formula of ion	m/z [theoretical]	Error [ppm]	Adduct (ion)	RT [min]	Molecular formula
1	191.0576	Quinic acid	$C_7H_{11}O_6$	191.0561	8.0	$[M-H]^-$	1.0	$C_7H_{12}O_6$
2	169.0146	Gallic acid	$C_7H_5O_5$	169.0142	-2.2	$[M-H]^-$	1.4	$C_7H_6O_5$
3	353.0875	Chlorogenic / Neochlorogenic acid	$C_{16}H_{17}O_9$	353.0878	-0.9	$[M-H]^-$	7.0	$C_{16}H_{18}O_9$
4	761.1361	Theasinesin B / Prodelphinidin-*O*-gallate	$C_{37}H_{29}O_{18}$	761.1359	-0.2	$[M-H]^-$	7.2	$C_{37}H_{30}O_{18}$
5	305.0668	Gallocatechin / Epigallocatechin	$C_{15}H_{13}O_7$	305.0667	0.4	$[M-H]^-$	7.4	$C_{15}H_{14}O_7$
6	337.0928	*p*-Coumaroylquinic acid (isomer I)	$C_{16}H_{17}O_8$	337.0929	-0.3	$[M-H]^-$	7.6	$C_{16}H_{18}O_8$
7	337.0926	*p*-Coumaroylquinic acid (isomer II)	$C_{16}H_{17}O_8$	337.0929	0.9	$[M-H]^-$	8.2	$C_{16}H_{18}O_8$
8	337.0928	*p*-Coumaroylquinic acid (isomer III)	$C_{16}H_{17}O_8$	337.0929	-0.3	$[M-H]^-$	8.6	$C_{16}H_{18}O_8$
9	289.0719	Catechin	$C_{15}H_{13}O_6$	289.0718	0.3	$[M-H]^-$	7.7	$C_{15}H_{14}O_6$
10	289.0717	Epicatechin	$C_{15}H_{13}O_6$	289.0718	-0.3	$[M-H]^-$	8.2	$C_{15}H_{14}O_6$
11	595.1665	Eriocitrin	$C_{27}H_{31}O_{15}$	595.1668	-0.5	$[M-H]^-$	7.7	$C_{27}H_{32}O_{15}$
12	353.0878	Chlorogenic / Neochlorogenic acid	$C_{16}H_{17}O_9$	353.0878	-0.1	$[M-H]^-$	7.8	$C_{16}H_{18}O_9$
13	593.1515	Epicatechin digallate / Kaempferol rutinoside / Kaempferol galactosyl rhamnoside / unknown conjugate	$C_{27}H_{29}O_{15}$	593.1512	-0.4	$[M-H]^-$	8.0	$C_{27}H_{30}O_{15}$
14	593.1512	Epicatechin digallate / Kaempferol rutinoside / Kaempferol galactosyl rhamnoside / unknown conjugate	$C_{27}H_{29}O_{15}$	593.1512	0	$[M-H]^-$	8.6	$C_{27}H_{30}O_{15}$
15	593.1514	Epicatechin digallate / Kaempferol rutinoside / Kaempferol galactosyl rhamnoside / unknown conjugate	$C_{27}H_{29}O_{15}$	593.1512	0.3	$[M-H]^-$	9.2	$C_{27}H_{30}O_{15}$
16	593.1511	Epicatechin digallate / Kaempferol rutinoside / Kaempferol galactosyl rhamnoside / unknown conjugate	$C_{27}H_{29}O_{15}$	593.1512	-0.2	$[M-H]^-$	9.4	$C_{27}H_{30}O_{15}$
17	563.1405	Catechin/Epicatechin glycoconjugate	$C_{26}H_{27}O_{14}$	563.1406	-0.2	$[M-H]^-$	8.4	$C_{26}H_{28}O_{14}$
18	479.0832	Myricetin-3-*O*-galactoside/glucoside	$C_{21}H_{19}O_{13}$	479.0831	0.3	$[M-H]^-$	8.6	$C_{21}H_{20}O_{13}$
19	771.1991	Quercetin based triglycoside	$C_{33}H_{39}O_{21}$	771.1989	-0.3	$[M-H]^-$	8.6	$C_{33}H_{40}O_{21}$
20	755.2040	Quercetin based triglycoside (deoxyhexose-hexose-deoxyhexose)	$C_{33}H_{39}O_{20}$	755.2040	0	$[M-H]^-$	8.8	$C_{33}H_{40}O_{20}$
21	755.2046	Kaempferol based triglycoside	$C_{33}H_{39}O_{20}$	755.2040	0.8	$[M-H]^-$	9.0	$C_{33}H_{40}O_{20}$
22	273.0767	Afzelechin / Epiafzelechin	$C_{15}H_{13}O_5$	273.0768	-0.4	$[M-H]^-$	8.8	$C_{15}H_{14}O_5$
23	609.1459	Quercetin diglycoside (hexose-deoxyhexose)	$C_{27}H_{29}O_{16}$	609.1461	-0.3	$[M-H]^-$	8.9	$C_{27}H_{30}O_{16}$
24	739.2090	Kaempferol triglycoside (deoxyhexose-hexose-deoxyhexose)	$C_{33}H_{39}O_{19}$	739.2091	0.1	$[M-H]^-$	9.0	$C_{33}H_{40}O_{19}$
25	431.0981	Vitexin / Isovitexin	$C_{21}H_{19}O_{10}$	431.0984	-0.6	$[M-H]^-$	9.0	$C_{21}H_{20}O_{10}$

26	441.0827	Epicatechin/Catechin gallate	$C_{22}H_{17}O_{10}$	441.0827	0.1	$[M-H]^-$	9.2	$C_{22}H_{18}O_{10}$
27	463.0878	Quercetin hexoside	$C_{21}H_{19}O_{12}$	463.0882	0.8	$[M-H]^-$	9.2	$C_{21}H_{20}O_{12}$
28	447.0931	Kaempferol hexoside	$C_{21}H_{19}O_{11}$	447.0933	0.3	$[M-H]^-$	9.5	$C_{21}H_{20}O_{11}$
29	447.0928	Kaempferol hexoside	$C_{21}H_{19}O_{11}$	447.0933	-1.1	$[M-H]^-$	9.7	$C_{21}H_{20}O_{11}$
30	516.1371	Quercetin based glycoconjugate	$C_{47}H_{52}O_{26}$	516.1379	-1.6	$[M-2H]^{2-}$	10.5	$C_{47}H_{54}O_{26}$
31	1033.2831	Quercetin based glycoconjugate	$C_{47}H_{53}O_{26}$	1033.2831	-0.1	$[M-H]^-$	10.5	$C_{47}H_{54}O_{26}$
32	450.1161	Quercetin based acylated triglycosiede	$C_{42}H_{44}O_{22}$	450.1168	1.6	$[M-2H]^{2-}$	10.7	$C_{42}H_{46}O_{22}$
33	901.2405	Quercetin based acylated triglycosiede	$C_{42}H_{45}O_{22}$	901.2408	0.4	$[M-H]^-$	10.7	$C_{42}H_{46}O_{22}$
34	442.1184	Catechin/Epicatechin based glycoconjugate	$C_{42}H_{44}O_{21}$	442.1193	-2.1	$[M-2H]^{2-}$	11.0	$C_{42}H_{46}O_{21}$
35	885.2453	Catechin/Epicatechin based glycoconjugate	$C_{42}H_{45}O_{21}$	885.2459	-0.7	$[M-H]^-$	11.0	$C_{42}H_{46}O_{21}$
36	715.1301	Theaflavin gallate	$C_{36}H_{27}O_{16}$	715.1305	0.5	$[M-H]^-$	11.5	$C_{36}H_{28}O_{16}$
37	715.1304	Theaflavin gallate	$C_{36}H_{27}O_{16}$	715.1305	-0.1	$[M-H]^-$	11.7	$C_{36}H_{28}O_{16}$
38	301.0351	Quercetin	$C_{15}H_{9}O_{7}$	301.0354	1.0	$[M-H]^-$	11.9	$C_{15}H_{10}O_{7}$
39	867.1432	Theaflavin digallate	$C_{43}H_{31}O_{20}$	867.1414	-2.1	$[M-H]^-$	12.1	$C_{43}H_{32}O_{20}$
40	285.0404	Kaempferol	$C_{15}H_{9}O_{6}$	285.0405	0.3	$[M-H]^-$	13.4	$C_{15}H_{10}O_{6}$
41	227.1286	dihydroxycarboxylic / dicarboxylic acid or esther	$C_{12}H_{19}O_{4}$	227.1289	1.2	$[M-H]^-$	15.7	$C_{12}H_{20}O_{4}$
42	227.1288	Acetylated hydroxycarboxylic acid	$C_{12}H_{19}O_{4}$	227.1289	-0.2	$[M-H]^-$	16.0	$C_{12}H_{20}O_{4}$
43	293.1759	Unknown carboxylic acid or esther	$C_{17}H_{25}O_{4}$	293.1758	-0.4	$[M-H]^-$	16.4	$C_{17}H_{26}O_{4}$
44	563.1192	Theaflavin	$C_{29}H_{23}O_{12}$	563.1195	0.6	$[M-H]^-$	11.2	$C_{29}H_{24}O_{12}$
Positive ion mode								
No.	m/z [measured]	Identification	Molecular formula of ion	m/z [theoretical]	Error [ppm]	Adduct (ion)	RT [min]	Molecular formula
1	175.1083	Theanine	$C_{7}H_{15}N_{2}O_{3}$	175.1077	3.4	$[M+H]^+$	1.0	$C_{7}H_{14}N_{2}O_{3}$
2	195.0886	Caffeine	$C_{8}H_{11}N_{4}O_{2}$	195.0877	4.8	$[M+H]^+$	7.7	$C_{8}H_{10}N_{4}O_{2}$
3	130.0864	Cycloleucine	$C_{6}H_{12}NO_{2}$	130.0863	1.0	$[M+H]^+$	1.3	$C_{6}H_{11}NO_{2}$
4	166.0865	Phenylalanine	$C_{9}H_{12}NO_{2}$	166.0863	-1.5	$[M+H]^+$	1.3	$C_{9}H_{11}NO_{2}$
